# Immune and Smooth Muscle Cells Interactions in Atherosclerosis: How to Target a Breaking Bad Dialogue?

**DOI:** 10.3389/fphar.2019.01276

**Published:** 2019-11-22

**Authors:** Damien Ramel, Stéphanie Gayral, Marie-Kerguelen Sarthou, Nathalie Augé, Anne Nègre-Salvayre, Muriel Laffargue

**Affiliations:** Department of Vascular Biology of the Institute of Metabolic and Cardiovascular Diseases (I2MC), Université de Toulouse 3, Institut National de la Santé et de la Recherche Médicale (INSERM) UMR1048, Toulouse, France

**Keywords:** inflammation, atherosclerosis, smooth muscle cells, therapeutic targets, cardiovascular diseases

## Abstract

Inflammation is a well-known pathophysiological factor of atherosclerosis but its therapeutic targeting has long been ignored. However, recent advances in the understanding of the immune mechanisms implicated in atherosclerosis have unveiled several therapeutic targets currently undergoing clinical trials. These studies have also shed light on a dialogue between the immune compartment and vascular smooth muscle cells (VSMCs) that plays a critical role in atherosclerotic disease initiation, progression, and stabilization. Our review focuses on the link between cellular and soluble immune effectors and VSMC behavior at different phases of the pathology. Furthermore, we discuss the potential targeting of these interactions to efficiently prevent cardiovascular diseases.

## Molecular and Cellular Determinants of Atherosclerosis

Cardiovascular diseases (CVDs) remain the leading cause of death worldwide and are in constant increase in western as well as low and middle-income countries [https://www.who.int/news-room/fact-sheets/detail/cardiovascular-diseases-(cvds)], underpinning the importance of finding novel therapeutic approaches.

The major cause of CVD is atherosclerosis. This pathology involves inflammatory and fibroproliferative mechanisms engaging communication between vascular cells [endothelial cells and vascular smooth muscle cells (VSMCs)] and immune cells. Atherosclerotic plaque progression leads to intimal thickening and culminates in late stages with plaque rupture which can cause myocardial infarctions and strokes.

The first initiating event of atherosclerosis involves shear stress perturbations responsible for endothelial cell dysfunction and inflammation leading to accumulation of low density lipoprotein (LDL) in the subendothelial space. Novel recent data has demonstrated that contrary to what was expected, this mechanism is not a passive movement of LDL across the endothelial barrier. Indeed, it involves the binding of LDL to the SRB-I receptor and subsequent transcytosis involving the guanine nucleotide exchange factor (DOCK4)/Rac pathway (Huang et al., 2019) and specific lipidic compartments (caveolae) found in great quantity in atheroprone regions. This mechanism is responsible for the initiation of the flow-dependent inflammatory priming of cells in atheroprone locations (Ramirez et al., 2019). LDL accumulation and oxidation in the medial area of arteries further amplifies inflammation by inducing chemokine secretion and expression of adhesion molecules at the surface of endothelial cells, such as intercellular adhesion molecule (ICAM) and vascular cell adhesion molecule (VCAM). This leads to a subsequent modification of the VSMC phenotype and the recruitment of immune cells.

The major involvement of VSMCs in atherosclerosis was revealed decades ago after observation that these cells were the main cellular component of atherosclerotic lesions (Parker, 1960; Imai et al., 1966). Since this discovery, decades of research have modulated our understanding of VSMC function during atherosclerosis. Importantly, recent studies have demonstrated that atherosclerosis development requires a dialogue between VSMCs, endothelial cells, and immune cells. Indeed, neutrophils, monocytes, lymphocytes, and mast cells are recruited to atherosclerotic lesions and interact with vascular cells. These interactions are critical as VSMCs undergo a phenotypic switching, the outcome of which depends on the immune environment.

## VSMC Plasticity During Atherosclerosis

In healthy conditions, VSMCs are mostly quiescent and differentiated, a phenotype called “contractile”. In this state, VSMCs express several markers such as 22 kDa actin-binding protein (SM22α/*tagln*), smooth muscle actin (αSMA) or smooth muscle cell myosin (SM-MHC/*myh11*). Furthermore, they ensure hemodynamic and structural regulation of the vessel wall (Lacolley et al., 2012; Bennett et al., 2016). In addition, VSMCs keep a high potential of dedifferentiation, in response to various external cues (growth factors, inflammation, matrix, lipoproteins, etc.) (Roostalu and Wong, 2018). Indeed, VSMCs are able to shift from a contractile phenotype to a so-called synthetic phenotype whereby cells are able to migrate, proliferate, and remodel the extracellular matrix. Phenotypic switching is characterized by (i) a progressive reduction or total loss of several VSMC lineage markers and (ii) increased proliferation capabilities associated with synthesis of extracellular matrix components and proteases (Ait-Oufella et al., 2006; Johnson et al., 2011; Newby, 2014). In the context of atherosclerosis, VSMC dedifferentiation can reach extreme phenotypes in which they are no longer identifiable as VSMCs. Dedifferentiated VSMCs can express macrophage markers such as CD11b, F4/80, or CD68 and acquire inflammatory cell properties by releasing pro-inflammatory cytokines or monocyte chemoattractants (Allahverdian et al., 2018). This VSMC-to-macrophage phenotypic switching implies genes such as KLF4 (Krüppel-like factor 4) (Alexander and Owens, 2012). Consistently, loss of KLF4 delays phenotypic switching or significantly reduces plaque size (Yoshida et al., 2008). This is associated with increased fibrous cap thickness which is indicative of higher plaque stability. Moreover, VSMC derived macrophage cells have the ability to accumulate lipids and become foam cells, a process which is also KLF4-dependent. A recent study has demonstrated that 60 to 70% of foam cells in mouse atherosclerotic lesions originate from VSMCs (Wang et al., 2019). These findings are consistent with those obtained in human atheromas and clearly indicate a major underestimated VSMC contribution to foam cell formation and atherosclerotic disease progression (Allahverdian et al., 2014). However, compared to classical monocytes, the phagocytic capabilities of VSMC-derived macrophages are reduced. Thus, these cells uptake less lipids as well as other materials such as dying cells or necrotic debris and participate by this way to the development of the necrotic core and intensify inflammation (Chaabane et al., 2014). Moreover, foam cell apoptosis leads to cholesterol deposition, ultimately forming the necrotic core. Amplification of the inflammatory process and growing of the necrotic core area is achieved by further VSMC and immune cell recruitment (Chaabane et al., 2014).

Hence, VSMC phenotypes conditioned by the immune environment (proliferation, apoptosis, matrix degradation, inflammation, or foam cell formation) are key determinants in the etiology of atherosclerosis leading either to a adingleleither to scl to sclerosis key determinants in the mainstability being responsible for plaque rupture and subsequent cardiovascular events.

## Immune Cells and Their Interaction With VSMCs During Atherosclerosis

**Figure 1:**
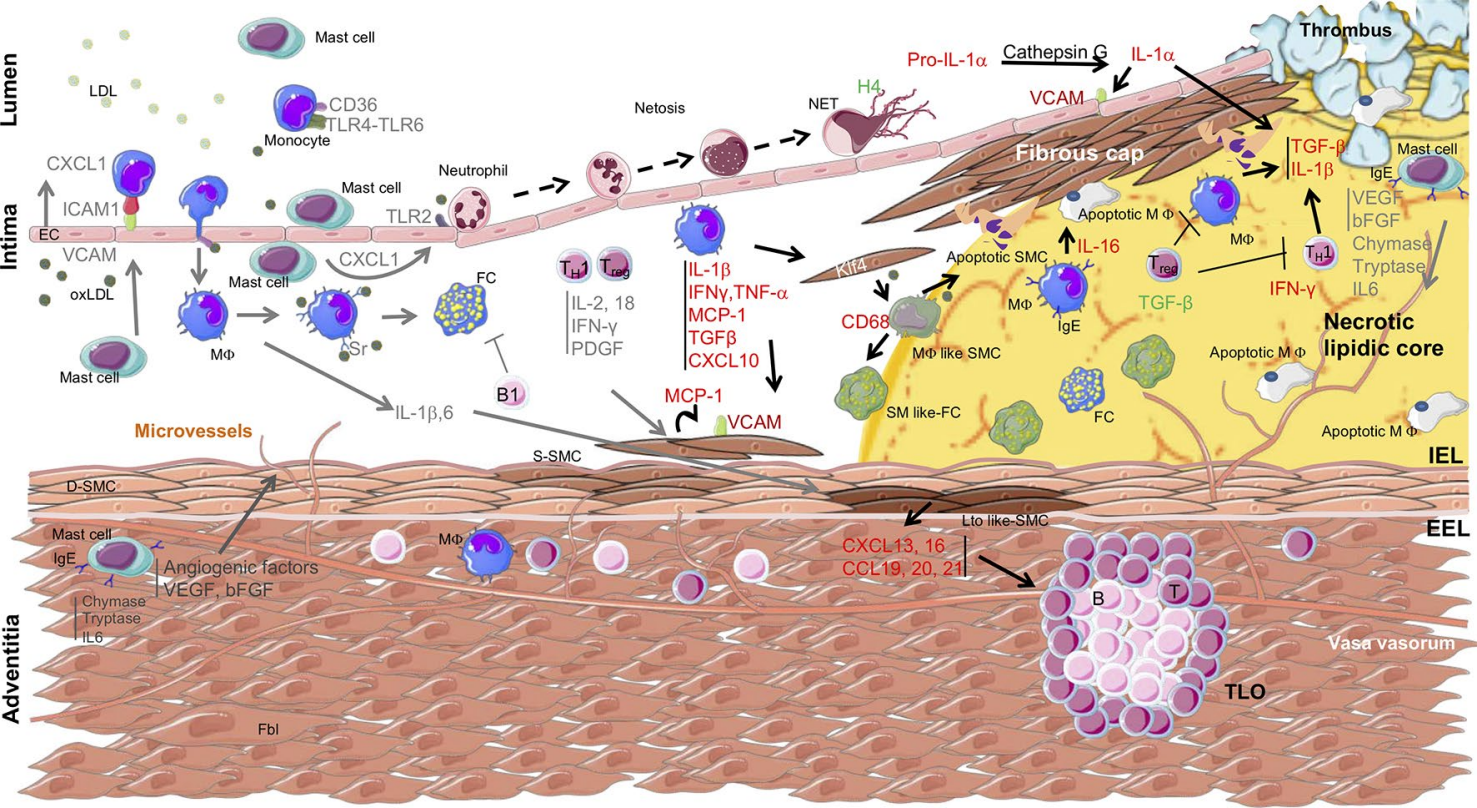
Overview of immune cell localization, cytokine secretion, and interaction with smooth muscle cells within the arterial wall during atherosclerosis progression. Arrows indicate the origin and target of immune mediators between the different cell types involved in atherosclerosis. The yellow area indicates the necrolipidic core. D-SMC, differentiated smooth muscle cell; S-SMC, synthetic smooth muscle cell; Lto like-SMC, tissue organizer-like smooth muscle cell; SM like-FC, smooth muscle like-foam cell; MΦ like SMC, macrophage like smooth muscle cell; apoptotic SMC, apoptotic smooth muscle cell; EC, epithelial cell; Fbl, fibroblast; TLO, tertiary lymphoid organ; IEL, internal elastic lamina; EEL, external elastic lamina; TC, lymphocyte T cell; BC, lymphocyte B cell; MΦ, macrophage; FC, foam cell; apoptotic MΦ, apoptotic macrophage; LDL, low-density lipoprotein; oxLDL, oxidized low-density lipoprotein; NETs, neutrophils extracellular traps; TLR, Toll-like receptor; CD, cluster of differentiation; KLF4, Kruppel-like factor 4; IgE, immunoglobulin type E; H4, histone H4; ICAM, intercellular adhesion molecule; VCAM, vascular cell adhesion molecule; IFN, interferon; PDGF, platelet-derived growth factor; MCP1, monocyte chemokine protein 1; TGF, transforming growth factor; CXCL, C-X-C motif chemokine; CCL, chemokine Ligands.

The first evidence of the role of inflammation in atherosclerosis was suggested in the 1980’s with the observation of inflammatory infiltrates in the coronaries of patients with unstable angina (Stratford et al., 1986; Wallsh et al., 1986) also observed in a rabbit experimental model fed a hypercholesterolemic diet (Schwartz et al., 1985). Over the years, numerous analyses have revealed the involvement of different immune cells in human atherosclerosis. The function of these immune cells has been clearly identified thanks to mouse models, such as LDLR^−/−^ and apolipoprotein E (ApoE)^−/−^ mice, that develop atherosclerosis (**Figure 1**).

### Monocytes/Macrophages

The real demonstration of the role of monocytes/macrophages was revealed by using M-CSF deficient mice crossed with ApoE^−/−^ or LDLR^−/−^(Qiao et al., 1997; Rajavashisth et al., 1998). These models presented a dramatic decrease in atherosclerosis development. Moreover, the recruitment of monocytes by their interaction with endothelial cells through adhesion molecules has been extensively described. VSMCs from healthy parts of the artery do not express adhesion molecules whereas ICAM-1, VCAM-1, and fractalkine (CXC3CL1) were found upregulated in VSMCs found in regions of atherosclerotic plaque (O’Brien et al., 1993; Endres et al., 1997; Braun et al., 1999; Barlic et al., 2007). These data suggest that synthetic VSMCs maintain monocytes/macrophages within the vessel through direct interaction between both cell types (Cai et al., 2004a; Cai et al., 2004b).

In fact, most of the communication between monocytes/macrophages and VSMCs has been shown to occur through immune mediators. Among them, interleukin 1β (IL-1β), tumor necrosis factor α (TNF-α), IL-6, and monocyte chemokine protein 1 (MCP-1) are highly secreted and play major roles in atherosclerosis, VSMC dysfunctions, and inflammation of the arterial wall (Bobryshev et al., 2016) (see **Table 1**). Nevertheless, in addition to their immune functions, these secreted molecules could also act on VSMC proliferation and survival. Therefore, targeting these molecules could have some deleterious effects on plaque stabilization. Blocking MCP-1, a chemokine also involved in VSMC proliferation and migration (Fougerat et al., 2012), has been demonstrated to be efficient to limit atherosclerosis progression and to increase plaque stability in the established atherosclerosis ApoE^−/−^ mouse model (Ni et al., 2001; Inoue et al., 2002). However, blocking IL-1β seems to interfere with smooth muscle viability modifying plaque composition (Gomez et al, 2018). Indeed, recent work done by Gomez et al. reported that IL1-ß produced by VSMCs in response to TNFα contribute to fibrous cap formation in advanced atherosclerotic lesions in ApoE-/- mice, hence stabilizing the atherosclerotic plaque. Importantly, while blocking IL-1β had no effect on lesion size, it completely inhibited beneficial outward remodeling (Warner and Libby, 1989; Gomez et al., 2018).

**Table 1:** List of major immune mediators impacting VSMC behavior during atherosclerosis.

Immune mediator	Cellular source	Major SMC behavior	References
IFN-γ	TH1	↗Proliferation	Lupieri et al., 2019
		CXCL10 secretion	
IgE	B cells	SMC apoptosis	Wang et al., 2011
MCP-1	Monocytes/macrophages, PDGF stim VSMC	↗Proliferation ↗Migration ↗Collagen	Ni et al., 2001; Inoue et al., 2002; Fougerat et al., 2012
TGF-β	Macrophage, Treg	↗Collagen	Mallat et al., 2001b; Lutgens et al., 2002; Robertson et al., 2003
IL-18	TH1	↘VSMC accumulation *in vivo*	Mallat et al., 2001a; Elhage et al., 2003
IL-6	Monocytes/macrophages	↗Proliferation	Bobryshev et al., 2016
TNFα	Monocytes/macrophages	↗Proliferation	Bobryshev et al., 2016
IL-1α	Neutrophils	↗Adhesion molecules expression	Folco et al., 2018.
Fractalkine	VSMC located in lesions	Interaction with monocytes	O’Brien et al., 1993; Barlic et al., 2007
IL-1β	Monocytes/macrophages	↗VSMC *in vivo*	Gomez et al., 2018
		↗collagen content *in vivo*	
NET, histone H4	Neutrophils	VSMC lysis	Folco et al., 2018; Silvestre-Roig et al., 2019
		Plaque destabilization	

IFN-γ, interferon γ; IgE, immunoglobulin type E; TGF-β, transforming growth factor β; IL, interleukin; MCP-1, monocyte chemokine protein 1; NETs, neutrophils extracellular traps; H4, histone H4; VSMC, vascular smooth muscle cell; PDGF, platelet-derived growth factor; CXCL, C-X-C motif chemokine.

In a similar manner, proteinases, secreted by macrophages, have been shown to have opposing roles in vascular remodeling and plaque rupture in advanced atherosclerosis (Newby, 2014). Indeed, it is clearly established that activation of metalloproteases is a prerequisite to induce matrix degradation, a process necessary to facilitate proliferation and migration of VSMC involved in vascular remodeling (Newby, 2005; Gerthoffer, 2007). Nevertheless, secretion of proteinases by macrophages during late atherosclerosis leads to destruction of extracellular matrix, associated in this case with plaque instability and plaque rupture (for review, see Newby, 2014). Thus, a better understanding of the basis for these opposing roles and a better characterization of the specific role of different proteinases needs to be done to propose effective therapies against myocardial infarction.

### Neutrophils

Adhesion molecules on endothelial cells also participate in the recruitment of neutrophils (Schmidt et al., 2016). Activation of Toll-like receptor 2 (TLR2) through binding of modified LDL has been proposed to play a role in endothelial cell-dependent inflammatory processes (Franck et al., 2017). TLR2 expression at the surface of endothelial cells has been correlated to neutrophil adherence in regions of local flow disturbance leading to superficial erosion (Franck et al., 2018). This supports a role for neutrophils in erosion-associated thrombosis. The activation of neutrophils promotes endothelial cell apoptosis and desquamation, favoring platelet recruitment, thrombin generation, and thrombus formation (Quillard et al., 2017). Moreover, neutrophil activation initiates a specific type of programmed cell death called NETosis which leads to the release of neutrophil extracellular traps (NETs). These are constituted of macromolecular aggregates containing DNA, histones, and granular enzymes. NETs could be responsible for thrombotic complications in mouse intimal lesions, recapitulating features of superficial erosion in humans (Franck et al., 2018). Moreover, exposure of human endothelial cells to NETs increases the expression of adhesion molecules such as ICAM and VCAM-1 as well as tissue factor (TF). Cathepsin G, a serine protease abundant in NETs, has been shown to cleave the pro–IL-1α precursor leading to the release of the more potent mature form of IL-1α responsible for ICAM-1, VCAM-1, and TF expression (Folco et al., 2018). In a model of advanced atherosclerosis with features of instability obtained by inducing a shear stress modifier around the carotid in high fat diet fed ApoE^−/−^ mice, the number of neutrophils was inversely correlated to the number of VSMCs and positively correlated with necrotic core area lesion size and plaque instability (Silvestre-Roig et al., 2019). In this work, a direct interaction between neutrophils and VSMCs has been described (Silvestre-Roig et al., 2019). The authors demonstrate that VSMCs found in the fibrous cap attract neutrophils, triggering the ejection of NET-like histone H4 which is responsible for VSMC lysis, ultimately leading to atheroma plaque destabilization. One question remains as to how neutrophils interact with VSMCs in the fibrous cap. One possibility proposed by (Silvestre-Roig et al., 2019) is that the release of cytotoxic NETs also induces endothelial cell death to favor the rapid infiltration of neutrophils within the fibrous cap.

### Mast Cells

Mast cells could also be involved in the recruitment of neutrophils and other leucocytes to the plaque by stimulating the upregulation of adhesion molecule expression in the endothelium and by secreting CXCL-1 (IL8) (Zhang et al., 2011; Wezel et al., 2015). These cells, first described in atherosclerotic plaques in 1954 (Cairns and Constantinides, 1954), play an important role in atherosclerosis development at different steps of the pathology. Indeed, they are able to take part in foam cell formation by increasing macrophage LDL uptake through granule secretion participating to the initiation of atherosclerosis (Kovanen, 1991). These cells, classically activated by immunoglobulin type E (IgE) through the FcεR1 receptor (i) secrete histamine which increases endothelial cell permeability (Kovanen and Bot, 2017) and (ii) produce a wide range of inflammatory cytokines involved in atherosclerosis initiation and progression (Sun et al., 2007). They are also able to secrete vascular endothelial growth factor and basic fibroblast growth factor, which participate to the formation of neo-vessels associated with plaque hemorrhage in complicated atherosclerosis (Kaartinen et al., 1996; Lappalainen et al., 2004). Mast cell number is significantly elevated in the shoulder region of coronary plaques susceptible to plaque rupture and thrombosis (Kaartinen et al., 1994), suggesting a role of activated mast cells in VSMC apoptosis. Consistent with this observation, it has been demonstrated that mast cell TLR4 activation is responsible for VSMC apoptosis through chymase and IL-6 release (den Dekker et al., 2012). Interestingly, it has also been demonstrated that the FcεR1 receptor is also expressed at the surface of VSMCs under inflammatory conditions. Its activation by IgE leads to cytokine secretion and SMC apoptosis (Wang et al., 2011) indicating that IgE could locally induce atherosclerotic plaque destabilization by a direct action on VSMCs or an indirect effect through mast cell activation.

### Lymphocytes

In addition to monocytes, neutrophils, and mast cells, lymphocytes are highly involved in atherosclerosis regulation. The adaptive immune system invades the atherosclerotic vascular wall from both the arterial lumen side and the adventitial side, playing an important role in immune-vascular cell dialogue during atherogenesis.

### T Cells

T cells have been found in human atherosclerotic plaques from the initiation phase to plaque rupture where they are found in a higher proportion (Otsuka et al., 2015). While the role of CD4^+^ T cells has been extensively studied, the role of CD8^+^ T cells is still unclear. Indeed, the genetic association of CVDs with CD8^+^ T cells has been described (Davies et al., 2012), however experimental studies in mice have reported contradictory results regarding CD8^+^ T cell functions (Zhou et al., 1996; Kyaw et al., 2013; van Duijn et al., 2019). Indeed, Kyaw et al. showed that CD8^+^ T cell depletion using a CD8α or CD8β monoclonal antibody ameliorated atherosclerosis in ApoE^−/−^ deficient mice fed a high-fat diet by reducing lipid and macrophage accumulation, apoptosis, necrotic cores, and inflammatory cytokines such as MCP-1, IL-1β, and interferon γ (IFN-γ). Conversely, a recent study performed by Van Duijn J et *al*. showed that in a mouse model of advanced atherosclerosis (high fat diet-fed LDLR^−/−^ mice), depletion of CD8 increased the Th1 CD4^+^ T cell fraction in lesions, resulting in increased inflammation and lesion destabilization (van Duijn et al., 2019). This discrepancy could be explained by the difference in the experimental protocol. Indeed, Kyaw et al. depleted CD8+ in 8 week old ApoE^−/−^ mice and then submitted the mice to a high fat diet. However, in their protocol Van Duijn J et al, used LDLR^−/−^ mice fed a high fat diet for 16 weeks and injected depleting antibodies during the last 6 weeks before sacrifice and atherosclerosis analysis (Kyaw et al., 2013; van Duijn et al., 2019). Therefore, further experiments need to be done to clearly define the role of CD8^+^ T cells during atherogenesis.

The role of CD4^+^ T cells depends on the subset concerned. It is now well established that Th1 cells secrete pro-inflammatory molecules such as IFN-γ which drives inflammation of the arterial wall (Frostegard et al., 1999; Smirnova et al., 2014), whereas Treg subpopulations dampen the immune system and decrease atherosclerosis development (Ait-Oufella et al., 2006). Consistently, Th1 cytokines are predominantly found in advanced human atherosclerotic plaque cells (Frostegard et al., 1999). Among the cytokines secreted by Th1 cells, IL-18 appears to negatively regulate VSMC accumulation within atheroma lesions in ApoE^−/−^ mice, supporting the notion that IL-18 is an important mediator of Th1-induced atherosclerosis (Mallat et al., 2001; Elhage et al., 2003). IFN-γ, the major Th1 cytokine also plays an important role in VSMC dysfunction. Indeed, IFN-γ positively contributes to VSMC proliferation in ApoE^−/−^ and LDLR^−/−^ mouse models (Gupta et al., 1997; Buono et al., 2003; Leon and Zuckerman, 2005). IFN-γ also contributes to plaque vulnerability by increasing matrix degradation through protease induction or by decreasing matrix synthesis by VSMCs. For example, IFN-γ increases the secretion of cathepsin S in human VSMCs which results in elastin degradation (Sukhova et al., 1998). IFN-γ could also favor the inflammatory response in VSMCs by increasing the expression of FcεR1, hence increasing VSMC ability to respond to IgE (Wang et al., 2011). Finally, IFN-γ directly acts on smooth muscle cells to induce the production of IFN-γ–inducible protein 10 (IP-10 or CXCL10), an important chemokine involved in atherogenesis and plaque destabilization (Heller et al., 2006; Segers et al., 2011). Interestingly, we recently demonstrated that this chemokine also acts directly on the endothelium to delay efficient healing after arterial injury (Lupieri et al., 2019). These data demonstrate a central role for VSMCs in relaying the T-cell response within the artery.

### B Cells

Studies to decipher the role of B cells in atherosclerosis carried out in the past years, controversies remain. These could be explained in part by the opposite functions of subsets of B cells (B1 and B2 cells) identified in human arteries (Tsiantoulas et al., 2015). B1 cells derived from fetal hematopoietic stem cells produce natural IgM independently of Th signals, and play a protective role in atherosclerosis (Lewis et al., 2009; Kyaw et al., 2011). Indeed, it has been proposed that IgM able to recognize oxidized phospholipids such as 1-palmitoyl-2-(5-oxovaleroyl)-sn-glycero-3-phosphorylcholine were produced during atherosclerosis. These antibodies impaired uptake of OxLDL by macrophages and recognized similar oxidation-specific epitopes on apoptotic cells in atherosclerotic lesions (Shaw et al, 2000). On the contrary, B2 cells produce immunoglobulins in response to Th signals which exert a pro-atherogenic action (Kyaw et al., 2010; Kyaw et al., 2012; Sage et al., 2012). Although atherosclerosis-promoting antibodies affect plaque composition and stability (Centa et al., 2019), an interaction between B cells and smooth muscle cells has not yet been reported.

### Tertiary Lymphoid Organs

The accumulation of lymphocytes can also be found in adventitial tertiary lymphoid organs (TLOs) which have been described in mouse and human atherosclerotic plaques. Cellular infiltration of the adventitia in human atherosclerotic arteries was first described by Schwartz et al. in 1962 (Schwartz and Mitchell, 1962). The comparative quantification of cellular infiltration in patients who suffered a myocardial infarction and in patients who died of non-cardiac causes demonstrated a possible correlation between adventitial immune cell infiltration and unstable coronary diseases (Kohchi et al., 1985). Cell aggregate organization in TLOs have been shown to develop in the abdominal aorta lamina adventitia of old ApoE^−/−^ mice, in association with atherosclerotic lesions. The evolution of TLOs has been classified into three different stages and varies from a structure predominantly composed of T cell aggregates (stage I) to a structure with separate T and B cell areas with ectopic germinal centers (stage III) (Grabner et al., 2009; Akhavanpoor et al., 2018). VSMCs are thought to play the role of non-hematopoietic stromal lymphoid tissue organizer-like cells (LTo), required for TLO formation (Lotzer et al., 2010). VSMCs switch to their LTo-like phenotype through the activation of their lymphotoxin β-receptor (LTβR) by a lymphocyte tissue inducer (LTi), like macrophages or immune cells of the intima plaque. LTo-like VSMCs secrete lymphorganogenic chemokines like CXCL13 or CCL21, attracting macrophages, dendritic cells, T cells, B cells leading to TLO organization in the adventitia (Moos et al., 2005; Hu et al., 2015; Srikakulapu et al., 2016). It seems that VSMCs may also contribute to the formation of TLOs *via* an LTβR-independent pathway. In this case, bone-marrow derived-macrophages play the role of LTi to trigger the production of CCL19, CCL20, and CXCL16 by VSMCs, promoting immune cell aggregation in the adventitia (Guedj et al., 2014).

Altogether, these data illustrate that the dialogue between immune cells and VSMCs (summarized in **Table 1** and **Figure 1**) must be taken into consideration to develop effective therapeutic approaches for treating atherosclerosis.

## Molecular Clues for Future Therapies

Current therapeutic strategies for atherosclerosis work by lowering cholesterol levels (statins, PCSK9 antibodies), reducing platelet functions, and controlling arterial tone (Zhao and Mallat, 2019). Nevertheless, atherosclerosis development is linked to important inflammatory processes of the arterial wall. Thus, targeting the immune compartment might be useful to fight CVDs and several clinical trials aiming at targeting immune processes have been done. However, to date, these trials were unsuccessful. Hypotheses to explain these adverse outcomes are multiple, including redundant inflammatory pathways or lack of functional data regarding the targeted pathways [reviewed in (Zhao and Mallat, 2019)]. Another possibility is that VSMC status can vary from one plaque to another. Thus, depending on their status, VSMCs might respond differently to a given therapeutic compound. Future therapeutic approaches will have to consider VSMC plasticity to improve their overall efficiency. Here, we will focus on the latest targets identified in clinical and pre-clinical studies that could impact VSMC behavior during atherosclerosis.

### Targeting IL-1β

The implication of the IL-1 pathway in atherosclerosis and VSMC proliferation and activation by inflammation has been extensively described. Numerous *in vivo* studies have demonstrated that inhibition of the NLRP3/IL-1β module decreases plaque development and deepens inflammation (Baldrighi et al., 2017). Altogether, these findings have opened the way to clinical trials targeting this pathway. Anti-IL-1β strategies have been studied in a phase III clinical study called CANTOS (Ridker et al., 2017). This study demonstrated that targeting IL-1β improves cardiovascular outcomes in patients with stable atherosclerosis. Nevertheless, this strategy failed to prevent cardiovascular events in high grade inflammatory patients and increased the number of fatal infections. This could be linked to the fact that the impact of IL-1β inhibition is still unclear. Recent *in vivo* evidence in ApoE^−/−^ mice indicates that IL-1β has atheroprotective functions. Indeed, Gomez et al. have clearly demonstrated that IL-1 signaling is required within VSMCs to prevent their apoptosis, retaining them in the fibrous cap in late stage atherosclerosis (Gomez et al., 2018). Thus, this therapeutic approach might indeed be deleterious and sheds light on VSMC plasticity in the different phases of atherosclerosis.

### Targeting Histone H4

In advanced atherosclerotic lesions, VSMC apoptosis is a hallmark of plaque rupture. One mechanism of VSMC death has been recently elucidated. Indeed, Silvestre-Roig et al. have reported that VSMCs are targeted by histone H4 containing NETs produced by infiltrated bone marrow derived neutrophils into the atheroma (Silvestre-Roig et al., 2019). Histone H4 molecules present at the NET surfaces interact with VSMC plasma membranes through electrostatic interactions and form pores inducing rapid cell death. Due to the importance of VSMC death in plaque stability, the authors developed a therapeutic strategy to prevent this histone H4-mediated effect. Using molecular dynamic simulation, they designed small peptides that disturb histone H4-membrane interactions. This analysis demonstrated that the N-terminal part of histone H4 is critical for membrane interactions. *In vitro*, the histone inhibitory peptide prevented histone H4 from interacting with VMSCs and protected VMSCs from cell death. *In vivo*, administration of this peptide using an osmotic mini-pump to mice carrying pre-existing atherosclerotic lesions (ApoE^−/−^ fed a high fat diet) increased VSMC number and consequently improved plaque stability. Thus, inhibition of histone H4 interactions with membranes could represent a potential therapeutic strategy for the prevention of advanced plaque rupture.

### Targeting CXCL10

C-X-C motif ligand 10 (CXCL10), or IP-10, is a small chemokine belonging to the CXC chemokine family (Luster and Ravetch, 1987). This chemokine mediates several biological functions in different cell types and tissues through binding to its receptor CXCR3. Of note, CXCL10 is responsible for monocyte and lymphocyte chemo-attraction to inflammatory sites. During atherosclerosis progression, endothelial cells, macrophages, and VSMCs express CXCL10 (van den Borne et al., 2014). Consistently, the ApoE^−/−^ mouse model in which CXCL10 or its receptor were invalidated displayed reduced atherosclerosis development (Veillard et al., 2005; Heller et al., 2006). This was also the case using a pharmacological inhibitor of CXCR3 (NBI-74330) in the LDLR^−/−^ mouse model (van Wanrooij et al., 2008). Altogether, these data place CXCL10 as an attractive target for atherosclerosis therapies (van den Borne et al., 2014). Interestingly, several monoclonal antibodies have been tested in phase II clinical trials for auto-immune diseases such as rheumatoid arthritis or ulcerative colitis. However, these antibodies demonstrated limited anti-inflammatory activity despite the major role of CXCL10 in inflammation (Yellin et al., 2012; Mayer et al., 2014). This divergence between clinical and experimental observations can be explained by several factors such as the differences in CXCR3 isoform expression between mice and humans. Indeed, humans express 3 isoforms, CXCR3-A, CXCR3-B, and CXCR3-alt (Lasagni et al., 2003; Ehlert et al., 2004) whereas mice only express one, CXCR3 closely related to CXCR3-A (Lu et al., 1999). Redundancy in the chemokine system could also explain these negative clinical results (Solari et al., 2015). Another factor of ineffective targeting is the amount of chemokines available for the antibodies. Indeed, chemokines such as CXCL10 are sequestered on glycosaminoglycans (GAGs). This interaction with GAGs prevents chemokine diffusion from the production site to the circulation, potentiating their local action. This observation suggests that higher doses of antibodies might be required to adequately inhibit free as well as GAG-trapped chemokines (Solari et al., 2015). However, in the diabetes RIP-LCMV glycoprotein mouse model, it has been shown that specific targeting of GAG-trapped CXCL10 was less effective in reversing hyperglycemia than antibodies directed against the free chemokine (Bonvin et al., 2017). Thus, further work is required to understand the molecular mechanism underlying CXCL10 functions in atherosclerosis, especially the implication of GAG-trapped versus free CXCL10. Interestingly, we recently demonstrated that after arterial mechanical injury, CXCL10 produced by VSMC in response to T-lymphocyte secreted IFN-γ directly inhibits endothelial healing. Thus, IFN-γ/CXCL10 axis may provide novel strategies to promote endothelial healing and prevent further atherosclerosis complications after therapeutic interventions (Lupieri et al., 2019).

### Targeting the PI3Kγ Signaling Pathway

In contrast to the other PI3K family members which are ubiquitously expressed, PI3Kγ presents a selective expression profile restricted to the hematopoietic and cardiovascular systems. Historically, PI3Kγ was first described for its role in leucocyte functions (Hirsch et al., 2000; Hawkins and Stephens, 2015). Later, we demonstrated that PI3Kγ drives immune-inflammatory processes within the arterial wall leading to atherosclerosis and restenosis (Fougerat et al., 2008; Fougerat et al., 2012; Smirnova et al., 2014). Moreover, PI3Kγ plays a role in VSMCs by being implicated in their migration (Fougerat et al., 2012). Finally, this kinase participates in the VSMC-immune dialogue by acting as a relay between T lymphocytes and endothelial cells during post-injury arterial healing (Lupieri et al., 2019). Thus, PI3Kγ represents an attractive therapeutic target and its inhibition could have a double beneficial impact on atherosclerosis by preventing VSMC phenotypic switching and accelerating endothelial healing. Interestingly, PI3Kγ inhibition is currently being tested in a clinical trial for solid cancers with the assumption that its immunomodulation functions in macrophages could exacerbate anti-tumoral immunity (De Henau et al., 2016; Kaneda et al., 2016). Depending on the upcoming results, such a therapeutic strategy could be considered in the context of CVDs.

## Concluding Remarks

Since the discovery of VSMC involvement in atherosclerosis, our understanding of how VSMCs contribute to this disease has evolved dramatically. Pioneering studies assumed that VSMCs exert a protective effect against plaque rupture by forming the fibrous cap. In the past decade, this role has been re-evaluated as these cells present high plasticity and contribute to different plaque phenotypes. In this context, communication between immune cells and VSMCs has been shown to play a crucial role in the modulation of VSMC behavior, promoting either plaque stability, progression, or rupture in response to specific stimuli. This aspect of VSMC biology should be investigated more deeply to be able to propose novel therapeutic avenues to fight against CVD.

## Author Contributions

DR, SG, AN-S, and ML originally conceived and wrote the manuscript. M-KS and NA designed figures and figure legends. All authors read and approved the final manuscript.

## Funding

This work was supported by grants from INSERM, Fondation de France to ML (Grant 201600066679) and to SG (Grant 0096285), Fondation Recherche Medicale “Physiopathologie Cardiovasculaire 2017” to ML, and ANR-ERA-CVD (JTC2017 PROACT) to ML. M-KS is supported by fellowship from Fondation Recherche Medicale.

## Conflict of Interest

The authors declare that the research was conducted in the absence of any commercial or financial relationships that could be construed as a potential conflict of interest.
